# Describing the Individual Spore Variability and the Parameter Uncertainty in Bacterial Survival Kinetics Model by Using Second-Order Monte Carlo Simulation

**DOI:** 10.3389/fmicb.2020.00985

**Published:** 2020-05-19

**Authors:** Hiroki Abe, Kento Koyama, Kohei Takeoka, Shinya Doto, Shigenobu Koseki

**Affiliations:** Graduate School of Agriculture Science, Hokkaido University, Sapporo, Japan

**Keywords:** non-isothermal inactivation, quantitative microbial risk assessment, Weibull model, *Bacillus simplex*, Monte Carlo simulation

## Abstract

The objective of this study was to separately describe the fitting uncertainty and the variability of individual cell in bacterial survival kinetics during isothermal and non-isothermal thermal processing. The model describing bacterial survival behavior and its uncertainties and variabilities during non-isothermal inactivation was developed from survival kinetic data for *Bacillus simplex* spores under fifteen isothermal conditions. The fitting uncertainties in the parameters used in the primary Weibull model was described by using the bootstrap method. The variability of individual cells in thermotolerance and the true randomness in the number of dead cells were described by using the Markov chain Monte Carlo (MCMC) method. A second-order Monte Carlo (2DMC) model was developed by combining both the uncertainties and variabilities. The 2DMC model was compared with reduction behavior under three non-isothermal profiles for model validation. The bacterial death estimations were validated using experimentally observed surviving bacterial count data. The fitting uncertainties in the primary Weibull model parameters, the individual thermotolerance heterogeneity, and the true randomness of inactivated spore counts were successfully described under all the iso-thermal conditions. Furthermore, the 2DMC model successfully described the variances in the surviving bacterial counts during thermal inactivation for all three non-isothermal profiles. As a template for risk-based process designs, the proposed 2DMC simulation approach, which considers both uncertainty and variability, can facilitate the selection of appropriate thermal processing conditions ensuring both food safety and quality.

## Introduction

Although thermal inactivation is the most often used procedure for controlling microbial contamination in processed foods, thermal processing at higher temperatures or longer heating times can induce chemical and physical deterioration in foods (Awuah et al., 2007; Fellows, 2009; Ling et al., 2015). The demand for food processing has begun to exceed the fundamental requirements of safety and shelf life, with more emphasis being placed on comprehensively labeled, high-quality, value-added foods that are convenient to consume (Awuah et al., 2007). In line with these goals, the microbial inactivation process in foods should be minimized. While thermal sterilization process is required for shelf-stable foods, milder thermal processing (<100°C) allowing for higher food quality retention is usually applied to ready-to-eat (RTE) food products intended to be stored under refrigeration. In recent years, many RTE foods, which maintain their quality through relieved long-time thermal inactivation higher than 80°C but below 100°C and storage at refrigeration temperatures, have been introduced. These products are called long-life refrigeration foods. However, some bacterial species are known to grow even at refrigeration temperatures; in particular, spore-forming spoilage bacteria such as *Bacillus simplex* can germinate and grow at refrigeration temperatures (below 10°C) and also have greater heat resistance than vegetative bacterial cells (Kobayashi et al., 2016). Moreover, some of *Bacillus* spp. including *B. simplex* can produce heat-stable toxins similar to cereulide (Taylor et al., 2005). The control of spore-forming bacteria can therefore help ensure food safety and quality of minimally processed RTE foods preserved at refrigeration temperatures.

Appropriate predictions and assessments based on mathematical models is necessary for food safety assurance. Many mathematical models have been used to predict bacterial behavior in foods and ensure food safety through quantitative microbial risk assessment (QMRA). However, one of the most popular models, the log-linear approach based on the D-value (decimal reduction time), can lead to over- or underestimation of the thermal death time (Peleg, 2006). To determine safe minimum processing levels, more accurate mathematical models for describing bacterial behavior during inactivation are needed.

The importance of uncertainty and variability in the evaluation of microbial behavior for QMRA purposes has been increasingly recognized (Nauta, 2000; Hoornstra and Notermans, 2001; Membré et al., 2006; FAO/WHO, 2008; Pouillot and Delignette-Muller, 2010; Koutsoumanis and Aspridou, 2017; Besten et al., 2018) In this context, “uncertainty” represents the lack of perfect knowledge of a parameter value, which might be reduced by further measurements, whereas “variability” represents the true randomness or heterogeneity of the population or environments, which are a consequence of the physical system and irreducible through additional measurements (Nauta, 2000). Because conventional kinetic models do not take into account uncertainty or variability between individual bacterial cells (Koutsoumanis and Aspridou, 2017; Besten et al., 2018), the estimation of bacterial behavior using kinetic modeling is considered insufficient in certain food safety management approaches (Koutsoumanis and Angelidis, 2007; Couvert et al., 2010). To describe variability and uncertainty in predictive modeling, stochastic models with Monte Carlo (MC) simulation using repeated random number simulations have been developed. As variability differs critically from uncertainty, the stochastic model can be roughly divided into sub-models for: (1) expressing variability, (2) expressing uncertainty, and (3) representing both.

Many studies have tried to express the variability of individual-cell inactivation behavior using stochastic process models (Aspridou and Koutsoumanis, 2015; Abe et al., 2019; Koyama et al., 2019a,b). Stochastic processes describe true randomness (true randomness comes even if completely same parameter value) with generating random number following a probability distribution. However, most such models, which describe bacterial reduction behavior at a constant temperature, cannot be adapted to the long come-up times frequently seen in the heating of actual food processings. In this study, it is tried to describe the variability in dead bacterial counts at a momentary dynamic condition during a short time lapse based on an individual bacteria’s death or survival model.

Parameter uncertainty has been investigated using bootstrap models to describe variations in bacterial behavior (Schaffner, 1994; Quinto et al., 2019). The bootstrap method (Efron and Tibshirani, 1991) is a resampling technique used to estimate statistics via computer simulation. Bootstrapping describes statistic parameters or values as distribution, and it could enable representing the variation in them caused from the lack of information by random resampling following observed values. In other words, bootstraps have been used to describe parameter uncertainty comes from variation of observed values, in terms of the lack of perfect knowledge of the parameter’s value (Nauta, 2000), as probability distributions.

Uncertainty and variability in bacterial behavior can be expressed using second-order Monte Carlo (2DMC) analysis and some researchers have advocated the application of 2DMC to describe both factors (Cassin et al., 1998; Wu and Tsang, 2004; Nauta et al., 2009; Pouillot and Delignette-Muller, 2010; Abe et al., 2019). The separation and combined description of uncertainty and variability play an important role in the valid prediction of bacterial behavior.

The objectives of this study were development and validation of a dynamic 2DMC model constructed by combining calculations of (i) uncertainty in model parameters, (ii) dynamic kinetics, and (iii) variability in individual cell inactivation times. The resulting dynamic model, which includes both uncertainty and variability, can contribute to the improvement of risk-based process design and the development of accurate risk assessment models.

## Materials and Methods

### Sample and Experimental Description

#### Bacterial Strain and Sporulation Conditions

The bacterial strain used as a model bacterium in this study was a *Bacillus simplex* isolate (isolation number: 2501), a spore-forming bacterial isolate associated with spoilage of refrigerated RTE food and provided by the Japan Canners Association (Tokyo, Japan). A sporulation procedure based on previous study (Kuroda et al., 2019) was applied. The frozen pure bacterial cultures were transferred to tryptic soy agar (TSA; Merck, Darmstadt, Germany) plate and incubated at 37°C for 5 days, after which an isolated colony of each bacterium was transferred to 5 mL of tryptic soy broth (TSB; Merck, Darmstadt, Germany) in a sterile plastic tube, which was then incubated at 37°C for 24 h. The cultures were transferred into soil extract agar (2 g/L beef extract, 3 g/L yeast extract, 10 g/L peptone, 5 g/L NaCl, 20 g/L agar, 1 g/L starch, 1 mL/L MnSO_4_ solution, 250 mL/L soil solution, 750 mL/L pure water) and the inoculated plates were again incubated at 37°C for 10 days. Following incubation, the bacterial colonies were scratched and collected using a platinum loop and suspended in 2 mL of 1/15 M phosphate buffer. Following confirmation of spore formation using phase contrast microscopy observations, the spores were collected by centrifugation (1,000 × *g* for 10 min at 20°C). The supernatant was discarded, and the spores were subsequently resuspended in 1/15 M phosphate buffer. This procedure was repeated three times and then the spore solutions were heated to 80°C for 10 min to remove remaining vegetative cells. The prepared spore suspensions were stored at −80°C and thawed gently (20 min in ice water) as needed.

#### Inactivation Trials for Model Fitting Dataset

**FIGURE 1:**
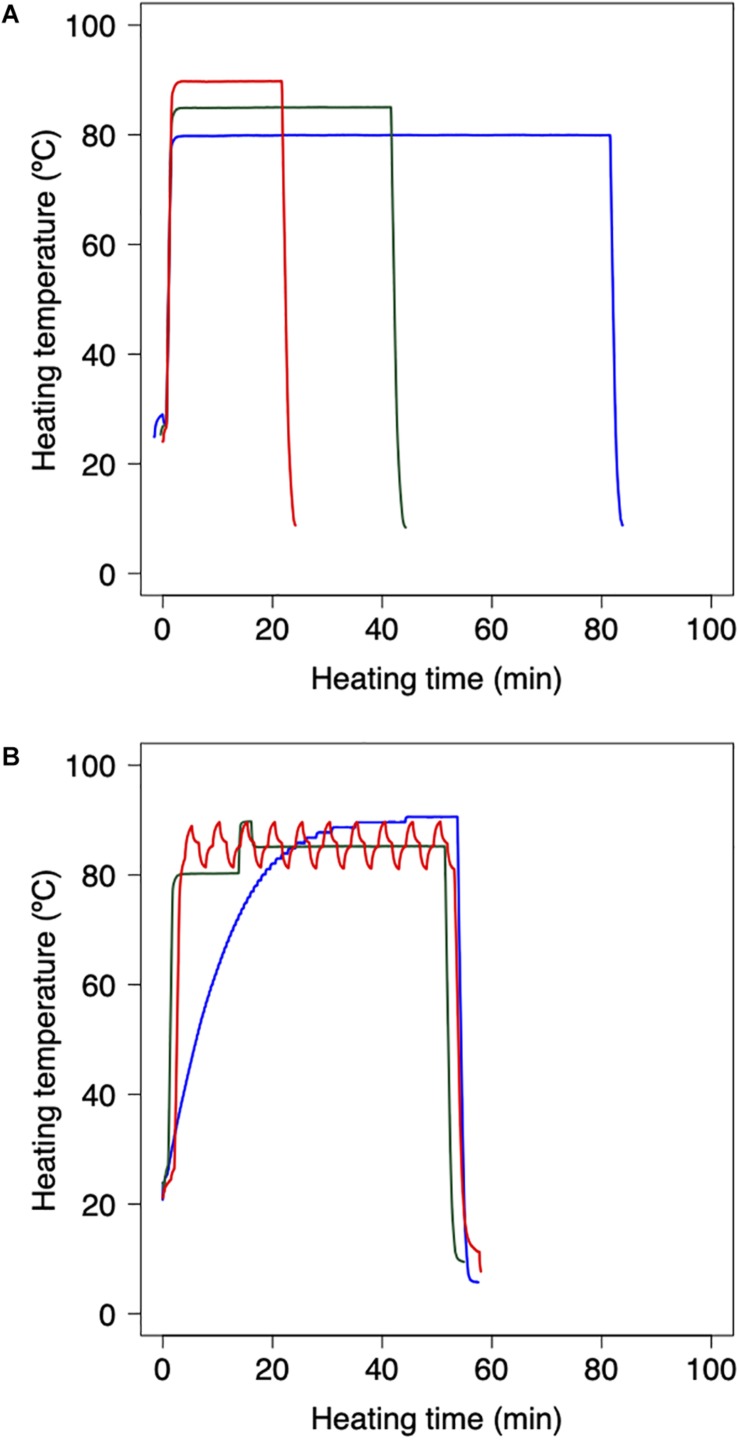
Example of heating protocol using thermal cycler. **(A)** Temperature profiles of inactivation trials for model fitting dataset. 80 min heating at 80°C (red), 35 min heating at 85°C (green), and 18 min heating at 90°C (blue). Preheating is conducted for 30 s at 25°C to standardize the initial temperature across trials. **(B)** Temperature profiles of inactivation trials for model validation dataset. Slow come-up (blue), bumpy (green) and waving (red) temperature profiles. Following the heating process, the 96-PCR microplates are immediately chilled at 4°C.

Bacterial reduction behaviors were experimentally observed using thermally processed *B*. *simplex*. Following the methodology applied in previous studies (Abe et al., 2018, 2019), the harvested *B*. *simplex* spores were heated using a thermal cycler and polymerase chain reaction (PCR) microplates. The *B. simplex* spores were washed by centrifugation and diluted with pH-adjusted TSB (pH:5.4, 5.8, 6.2, 6.6, and 7.0) and aliquots of diluted spore suspension (100 μl) were then dispensed into three representative wells of a 96-well PCR microplate to obtain cell concentrations of 10^6^ CFU/well. The temperature profile of the wells was checked beforehand to ensure that there were no temperature differences. Following 30 s of preheating at 25°C to standardize the initial temperature across the trials, the microplates were heated at various temperatures (80, 85, and 90°C) on a MiniAmp Plus Thermal Cycler (Thermo Fisher Scientific, Waltham, MA, United States). Immediately after heating, the PCR microplates were cooled to 4°C. Figure 1A shows examples of the thermal profiles in each one of these processes. The total duration of the trials depended on the temperature at various time intervals. Serial 10-fold dilutions of samples in TSB were plated onto TSA, and population survival was determined after a 24 h warming up of three replicate microplates to 37°C, a culturing condition that was previously confirmed as capable of assuring recovery.

#### Inactivation Trials for Model Validation Dataset

As a validation data set, *B. simplex* suspensions of 100 μL, which initial counts were 10^*n*^ CFU (*n* = 2, 3, and 4), were heated with the thermal cycler. Inactivation conditions were performed in three dynamic temperature profiles (slow-come-up, bumpy, and waving temperature profiles; Figure 1B), three pH conditions, and 10 repetitions per each heating time. To compare the simulated and the observed value, maximum RMSE (root mean square error) and minimum RMSE were derived from them.

### Estimation of Weibull Parameter Uncertainty and Thermotolerance Heterogeneity With Bootstrap Methods

#### Resampling Viable Bacterial Ratio Data

**FIGURE 2:**
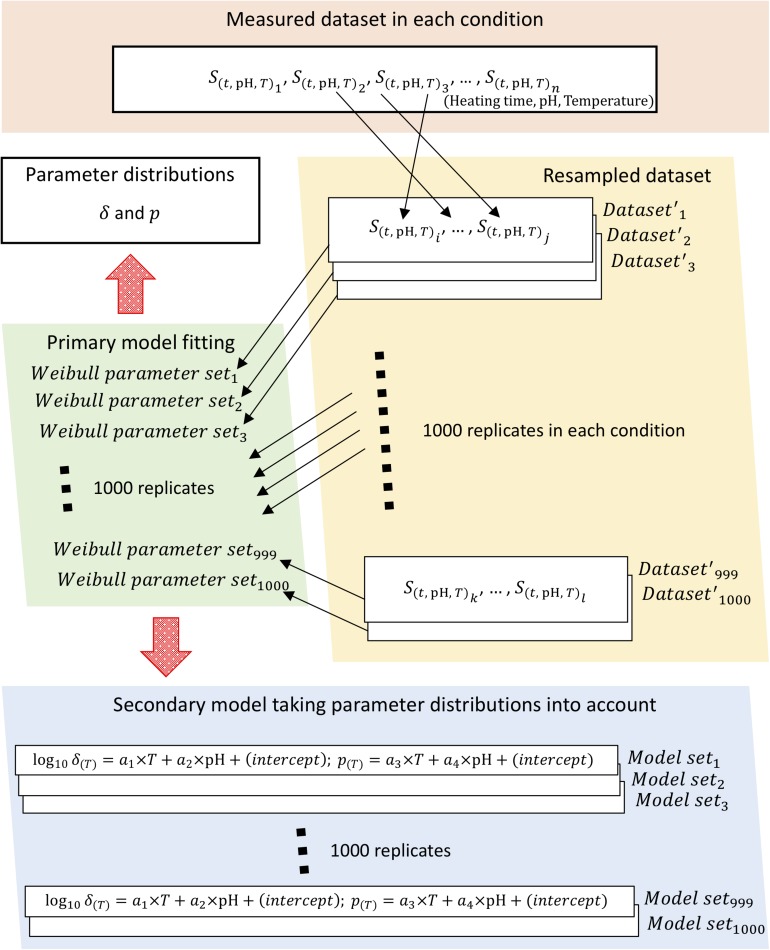
Schematic of the bootstrapping procedure. The bootstrapping was conducted using 1,000 replicates of both the Weibullian parameter distributions and the secondary models for each parameter, with the uncertainty of each parameter considered based on 1,000 replicates of the resampled surviving cell ratio.

To obtain parameter distributions of Weibull model, we described the parameter uncertainties and thermotolerance heterogeneity of individual cells using a non-parametric bootstrap. Figure 2 shows a schematic of the overall bootstrapping process applied in this study. To carry out bootstrapping, new samples are recollected from observed data (non-parametric bootstrapping) or from a fitted distribution (parametric bootstrapping). Non-parametric bootstrapping is useful when the distribution of a population is unknown, poorly understood, or when the investigator does not want to use a predefined population distribution; parametric bootstrapping is useful in problems in which some knowledge of the form of the underlying bacteria population is available and for comparison with non-parametric analysis (Quinto et al., 2019). In this study, we applied non-parametric methods to account for the uncertainties in observed values.

Sample sets of 1,000 iterations were resampled with replacement for Weibull fitting to calculate the random variables as Weibull model parameters. The change in surviving bacterial counts was represented in the form of a survival ratio,*S*_(*t*)_, defined as the ratio between the number of survivors after exposure time *t*, *N*_*(t)*_, and the initial population, *N*_*0*_, i.e., *S*_(*t*)_=*N*_(*t*)_/*N*_0_. Each condition (heating time, pH, heating temperature) was represented using three repetitions of observed data; in other words, the survival ratio data for three replicates were resampled three times, including duplication, per heating condition. Each set of three samples was randomly selected as resampled data from the set of data observed at all thermal conditions. Each resampled set was used as a replicate bootstrapped dataset, of which a total of 1,000 were generated.

#### Fitting Resampled Data of Survival Ratio to Weibull Model and Secondary Models

To perform Weibull fitting of the 1,000 bootstrapped data sets described in the previous section, the parameters of a Weibull model at each temperature were fitted to the respective values at each temperature of the resampled survival ratios derived from the inactivation experiment. The Weibull models were constructed under the assumption that vegetative cells and spores within a population have different resistances:

(1) $${\text{log}}_{10}\,S_{\left(t\right)}=-{\left(\frac{t}{\delta }\right)}^{p}$$

where δ and *p* are constant parameters of the Weibull modelfrom which the mode, mean, variance, and coefficient of skewness of the distribution can be calculated (Mattick et al., 2001). The Weibull model is one of the general mathematical models to describe various behaviors of inactivation or reduction (Davey, 1993; Mafart et al., 2002; Van Boekel, 2002; Peleg and Normand, 2004; Gomez et al., 2005; Koseki et al., 2015).

The δ is called the time of the first decimal reduction (time necessary to inactivate a decimal reduction of the microbial population when *p* parameter is 1.0) and is the so-called scale parameter; *n* and *p* parameter is the so-called shape parameter (Gomez et al., 2005). Its value depends on the shape of the survival curve; *p* < 1 for concave upward survival curves, *p* = 1 for linear survival curves and *p* > 1 for concave downward survival curves (Gomez et al., 2005). In this study, δ and *p* were used to describe the experimental data obtained from the kinetic experiments using non-linear regression analysis (least mean square error) in which 1,000 iterations of the resampled Weibullian parameters were estimated at each pH and temperature.

The parameters of the secondary models were then calculated based on the Weibullian parameters obtained for the primary model, with 1,000 replicates of the secondary model estimated to describe the relationship between pH, temperature, and the primary parameters derived in the preceding section. Based on the results of previous studies, the temperature dependency of the Weibull model parameters δ and p was described using the fitted exponential-type functions δ_*(T)*_ and *p*_*(T)*_, respectively, where δ_*(T)*_ is given as:

(2) $${\text{log}}_{10}\,\delta_{\left(T\right)}=a_{1}\times T+a_{2}\times \text{pH}+\left(i\,n\,t\,e\,r\,c\,e\,p\,t\right).$$

This interaction-term-free polynomial equation was initially proposed (Davey, 1993) to describe the combined effect of temperature and pH on the *D*-values of *Clostridium botulinum* spores (Gomez et al., 2005). It has been reported that *p*_*(T)*_ varies linearly or is constant at both increasing and decreasing temperatures (Van Boekel, 2002); in a constant case, it can be represented by a linear equation (when the coefficient of temperature and pH is 0). The following linearly expression for *p*_*(T)*_ was used in this study:

(3) $$p_{\left(T\right)}=a_{3}\times T+a_{4}\times \text{pH}+\left(i\,n\,t\,e\,r\,c\,e\,p\,t\right).$$

The coefficients of the parameters of each of the models were calculated using a multivariate linear least square error regression.

The convergences of parameter distributions were indicated by the Gelman-Rubin convergence statistic ($\hat{R}$-value). The $\hat{R}$-value is generally used for confirming the convergences of parameter distributions in Markov chain Monte Carlo (MCMC) on Bayesian statistic (Vehtari et al., 2019); it is said that $\hat{R}$-value lower than 1.1 indicates the convergence of the parameter distribution.

### Numerical Estimation for True Randomness Variability in Bacterial Behavior During Non-Isothermal Conditions With Stochastic Model

#### Numerical Calculation of Bacterial Non-Isothermal Inactivation for Stochastic Simulation

To serve as the basis of our dynamic probability model, we created a dynamic kinetic model based on previously developed methodologies (Campanella and Peleg, 2001; Peleg et al., 2005; Corradini and Peleg, 2009). Under fluctuating temperature conditions, a very short time interval was taken into consideration, [*t*_*i*_,*t*_*i*__+__1_]. The parameters of Weibullian model for inactivation rate during the interval can be assumed as average of the parameters of *t*_*i*_ and *t*_*i*__+__1_ (Peleg, 2006), following:

(4) $$\bar{\delta }=\frac{\delta_{t_{i}}+\delta_{t_{i+1}}}{2},\bar{p}=\frac{p_{t_{i}}+p_{t_{i+1}}}{2}.$$

The actual heating time is described by transforming Eq. 1 (Campanella and Peleg, 2001) as follows:

(5) $$t^{*}={\left[\frac{-\log_{10}S_{t_{i}}}{\bar{\delta }}\right]}^{\frac{1}{\bar{p}}}.$$

Since the parameters are constant values in the intervals, the reduction behavior can be assumed as the isothermal in the interval. Therefore, the survival ratio at *t*_*i+1*_, *S*_*t_i+1*_, can be described as following Eq. 6:

(6) $$\log_{10}S_{t_{i+1}}=-{\left(\frac{t^{*}+\Delta \,t}{\bar{\delta }}\right)}^{\bar{p}}=-{\left(\frac{{\left[\frac{-\log_{10}S_{t_{i}}}{\bar{\delta }}\right]}^{\frac{1}{\bar{p}}}+\Delta \,t}{\bar{\delta }}\right)}^{\bar{p}}.$$

$$\Delta \,t=t_{i+1}-t_{i}$$

Furthermore, the survival counts can be described by transformation of Eq. 6 following:

(7) $$N_{t_{i+1}}=N_{0}\times 10^{-{\left(\frac{t^{*}+\Delta \,t}{\bar{\delta }}\right)}^{\bar{p}}}.$$

Defining the dead bacterial count over the time interval Δ*t* as

(8) $$N_{d\,e\,a\,d\,c\,e\,l\,l\,\left[t_{i},t_{i+1}\right]}=N_{\left(t_{i}\right)}-N_{\left(t_{i+1}\right)},$$

We assumed these kinetic estimations as average behavior of stochastic behavior.

#### Stochastic Description of Variability in Viable Bacterial Counts

In this study, two types of variability were considered, namely the initial cell counts variability and the variability in cell inactivation during short time intervals, and were described by two types of probability distributions. According to previous studies (Koyama et al., 2016; 2019b; Abe et al., 2019), the initial cell counts variability can be described as a Poisson distribution. Therefore, the initial spore number was generated as a random number following a Poisson distribution with an average of *N*_*(0)*_, i.e.,

(9) $$N_{\left(0\right)}∼P\,o\,i\,s\,s\,o\,n\,\left(\bar{N_{0}}\right),$$

where *N*_*(0)*_ is the simulated initial cell count from a Poisson distribution in which the expected value is $\bar{N_{0}}$.

**FIGURE 3:**
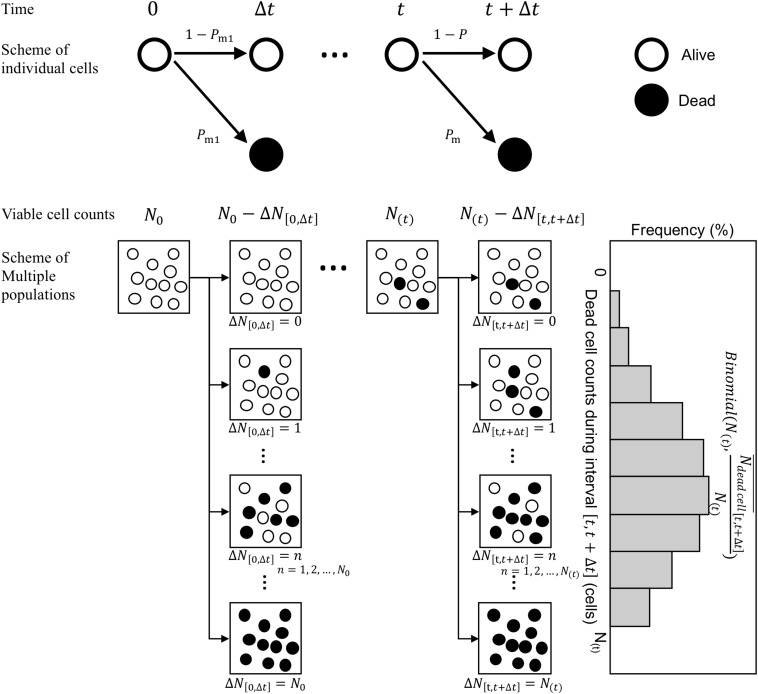
Schematic of the Markov Chain Monte Carlo (MCMC) procedure. The proposed stochastic explanation describes bacteria death or continued survival based on the pure death process in the branch of modeling biological populations.

Next, we consider the variability in bacterial death counts under heating process during the infinitesimal interval (Figure 3). A model of possible explanation of bacteria death or continued survival (Corradini et al., 2010) was used. Focusing on individual cells, a cell has a probability of *P*_*m*_ to be inactivated and 1−*P*_*m*_ to remain viable after a short time interval Δ*t*. Assuming that individual bacterial cells (or spores) get inactivated during this short time interval, the probability *p*_*(k)*_, i.e., the probability that *k* cells/spores are inactivated out of *N*_*(t)*_ surviving cells/spore, can be expressed as a binomial distribution. The general binomial distribution form is $p(k)\;\left({}_{k}^{n}\right)p^{k}(1-{p)}^{n-k}$ (Rychlik and Ryden, 2006) and the expected value of the general distribution is calculated as *np*. Here, the average value of the dead bacterial counts during an interval from *t* to *t* + Δ*t*, ${\bar{N_{d\,e\,a\,d\,c\,e\,l\,l}}}_{\left[t,t+\Delta \,t\right]}$, was assumed as the kinetic estimation. The binomial distributions are generally used as a probabilistic distribution describing the pure death process in the branch of modeling biological populations (Renshaw, 1993). ${\bar{N_{d\,e\,a\,d\,c\,e\,l\,l}}}_{\left[t,t+\Delta \,t\right]}$ follows a binomial distribution of size *N* with a probability parameter $\frac{{\bar{N_{d\,e\,a\,d\,c\,e\,l\,l}}}_{\left[t,t+\Delta \,t\right]}}{N_{\left(t\right)}}$. Thus, the simulated survival cell counts *N* at exposure time*t* can be described in terms of *N* as

(10) $$N_{\left(t+\Delta \,t\right)}∼N_{\left(t\right)}-B\,i\,n\,o\,m\,i\,a\,l\,\left(N_{\left(t\right)},\frac{{\bar{N_{d\,e\,a\,d\,c\,e\,l\,l}}}_{\left[t,t+\Delta \,t\right]}}{N_{\left(t\right)}}\right),$$

where $B\,i\,n\,o\,m\,i\,a\,l\,\left(N_{\left(t\right)},\frac{{\bar{N_{d\,e\,a\,d\,c\,e\,l\,l}}}_{\left[t,t+\Delta \,t\right]}}{N_{\left(t\right)}}\right)$ is a random number derived from a binomial distribution of size *N*_(_*_*t*_*_)_ in which the probability parameter is $\frac{{\bar{N_{d\,e\,a\,d\,c\,e\,l\,l}}}_{\left[t,t+\Delta \,t\right]}}{N_{\left(t\right)}}$.

### Second-Order Monte Carlo Simulation Procedure

Three types of randomness were used to describe variability and uncertainty under the proposed bacterial inactivation model. Under the first type, which describes the variability in the initial bacterial count. The second type describes the uncertainty in an estimated parameter derived from the secondary models obtained from the bootstrap. The third type describes true randomness variability in bacterial reduction using Eq. 10. Using these approaches, the specific uncertainty in bacterial behavior was simulated as follows:

1. Using the first approach, a simulated initial count,*N*_(0)_, was generated as a random number derived from the Poisson distribution. (Eq. 9):
2. To describe the uncertainty stemming from variation in the observed values, a secondary model set was randomly selected from the model set of 1,000 replicates obtained from the bootstrap.
3. The two Weibull parameters were derived from the secondary model set randomly selected in procedure II and the thermal history, respectively.
4. Based on the dynamic kinetic model (Eq. 8) with III’s parameters, the average inactivated spore count, ${\bar{N_{d\,e\,a\,d\,c\,e\,l\,l}}}_{\left[t,t+\Delta \,t\right]}$, during the very short time interval [*t*,*t* + Δ*t*] (Δ*t* was set as 1 s in this study) was obtained from the derived Weibull parameters and *N*, the surviving cell count at *t*.
5. From ${\bar{N_{d\,e\,a\,d\,c\,e\,l\,l}}}_{\left[t,t+\Delta \,t\right]}$, a simulated inactivated cell count during the time interval was generated as a random number, Δ*N*_[*t*,*t* + Δ*t*]_, derived from the binomial distribution.
6. A simulated survival cell count at *t* + Δ*t* was derived from the simulated inactivated cell count during the short time interval and *N*_(*t*)_ (Eq. 10).
7. Procedures III–VI were repeated until the thermal treatment had finished or *N*became zero.

The above series of procedures was repeated 100 times to determine the variance, including variability and one type of uncertainty, in bacterial behavior. The R statistics (Ver. 3.5.1 for Mac OS X) statistical software was used to carry out all statistical analyses.

## Results and Discussion

### Distribution of Weibullian Parameters Derived From the Bootstrap

**FIGURE 4:**
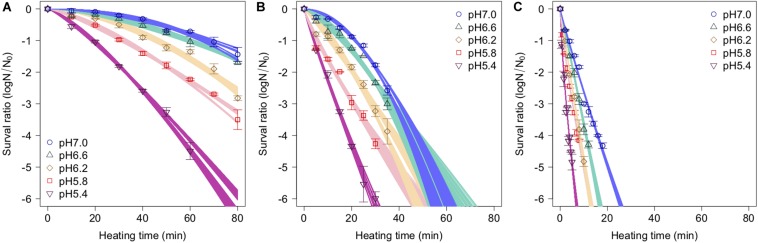
Survival kinetics of *Bacillus simplex* spores under different temperature conditions, **(A)** 80°C, **(B)** 85°C, and **(C)** 90°C. The points, error bars, and curves indicate, average value of observed survival ratio (three replicates), standard errors of observed survival ratio, and estimated Weibull model curves, respectively, based on 1,000 bootstrap replicates.

**FIGURE 5:**
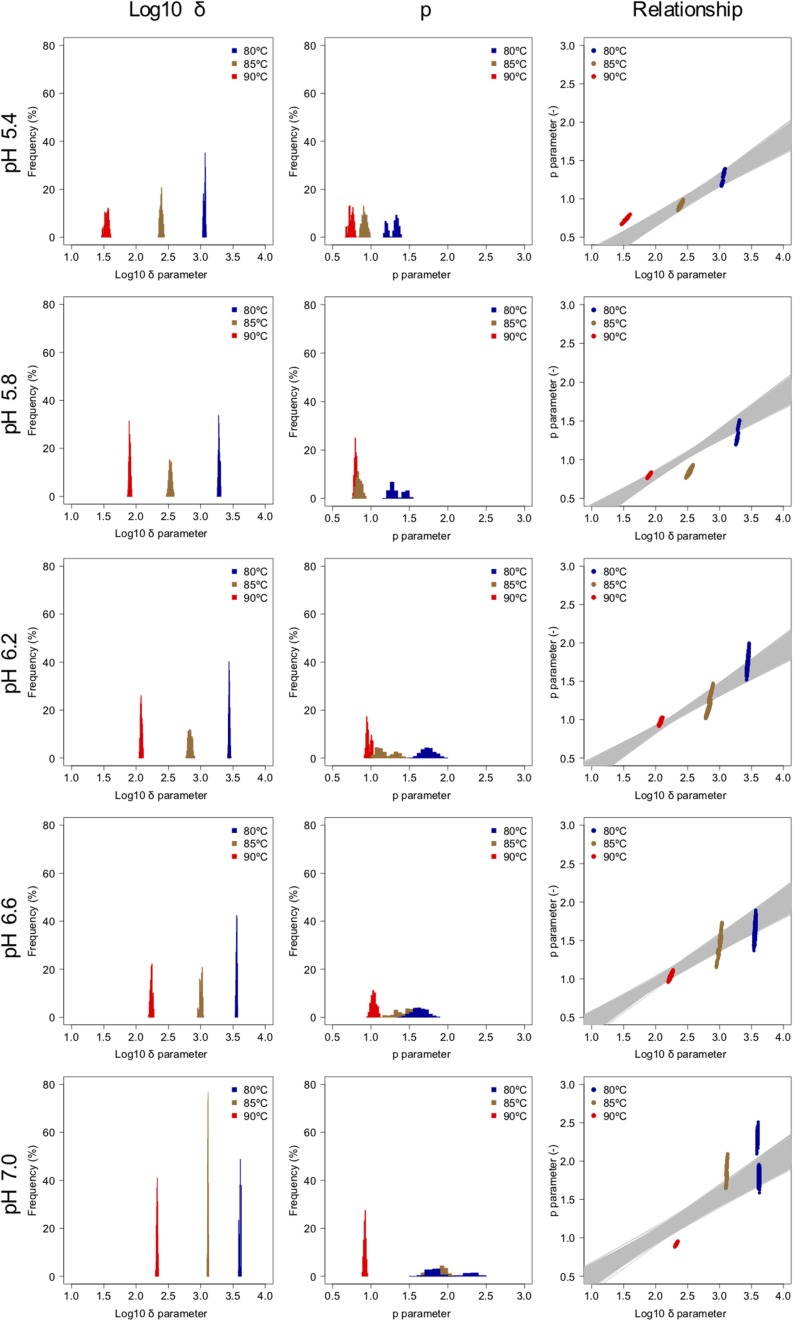
Weibullian parameter distributions under each condition derived from 1,000 bootstrap replicates, and the relationship between the two bootstrapped Weibullian parameters. The gray lines indicate the relationship between the two estimated parameters from bootstrapped secondary model sets.

The spore survival kinetics under heating for each condition and the Weibull fitted survival curve based on 1,000 bootstrap replicates are shown together in Figure 4, in which the error bars indicate the standard deviations (from three observations) under the respective conditions. Based on the variation in the estimated Weibullian model, each condition has 1,000 replicates. The estimated Weibull model parameters (i.e., log_10_δ and*p*) derived from the bootstrap were convergent, since all the $\hat{R}$-values of the parameters were equal to 1.0; they followed empirical distributions (Figure 5), with both decreasing as either the temperature increased, or the pH decreased. These distributions come from the variations in combinations of resampled observed values by the bootstrap. Therefore, it reflects the fitting uncertainty in Weibullian parameters and the thermotolerance variability of individual cells that arise from observations. The variation in *p* values was larger than that observed in log_10_δ. Under some conditions in which the variation in *p* was particularly large, its distribution had two peaks (Figure 5). These indicate that it is difficult to detect the true one parameter from observed bacterial survival kinetics during thermal inactivation.

**FIGURE 6:**
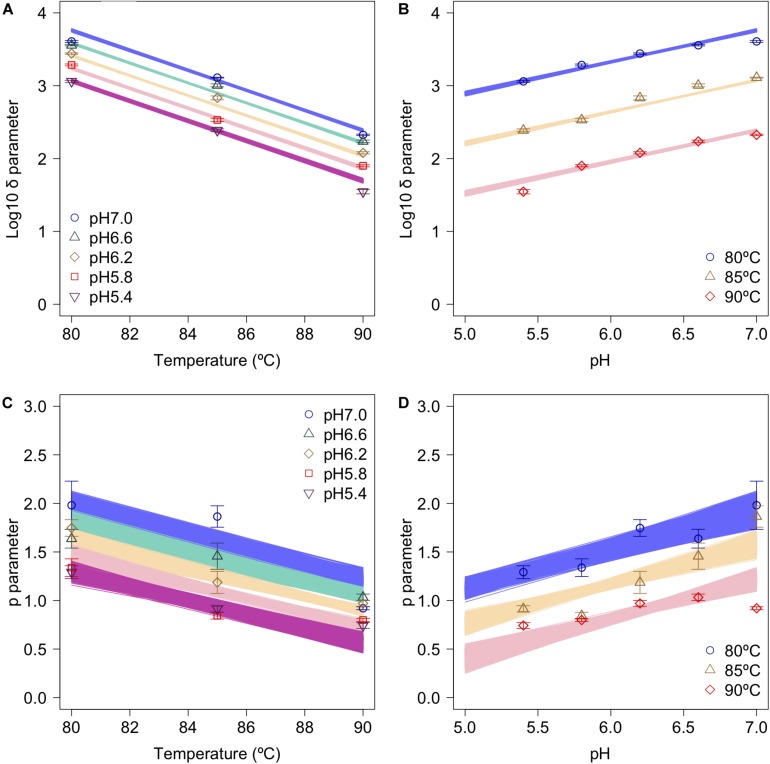
Changes in log_10_δ and *p* as functions of heating temperature and pH (data points). **(A)** log_10_δ against temperature; **(B)** log_10_δagainst pH; **(C)** *p* against temperature; and **(D)** *p* against pH. Error bars indicate standard deviation of the 1,000 estimated Weibullian parameter replicates; lines indicate secondary model (**A,B**: Eq. 2; **C,D**: Eq. 3) derived from 1,000 bootstrap replicates.

The secondary model was used to describe the relationship between the bootstrapped parameters and those for pH and temperature (Figure 6). In the figure, the points, error bars, and lines indicate the average values of the bootstrapped parameters, the standard deviations of the bootstrapped parameters, and the 1,000 replicates of the bootstrapped secondary model, respectively. It is seen that the secondary model has confirmed the results of previous studies (Mafart et al., 2002), in which the temperature dependencies of the δ parameter were shown to follow a conventional *D*-value form. The pH dependencies of δ also follow a conventional *D*-value form. Although it has been reported that *p* parameter does not show any temperature and/or pH dependency in most cases (Van Boekel, 2002), the *p* parameter values obtained in the present study show a temperature and pH dependency (Figures 6C,D). Similar to the present study, there are some reports that it was found that *p* decreased linearly as the temperature rose before reaching a constant level, suggesting that the results for *p* corresponded to a partially linear regression (Campanella and Peleg, 2001; Gomez et al., 2005).

Apart from temperature/pH dependency of the parameters (δand *p*), there is an interaction between the δ and *p* parameters, because the shapes depend on the both of parameters (Peleg, 2006). The developed 2DMC model enables to take into account the interaction of both the parameters, because it estimates the Weibull parameters using sets of secondary models of the corresponding δ and *p* parameters to resampled datasets. Comparing the fitted parameters’ interaction and the estimated parameters’ interaction by the bootstrapped secondary model sets, the bootstrapped secondary model sets explain linearly the interaction between δ and *p* parameter as shown in the right columns of Figure 5.

Comparing the variations in *p*-parameters and log_10_δ derived from the bootstrapped parameters, the variations in the estimated *p*-parameter derived from the secondary model using bootstrapping were larger than those of log_10_δ, possibly because they arose from small differences such as those between repetitions (Figure 6). These results confirm the difficulty to develop a secondary model of *p* from only one parameter per condition.

### Validation of Dynamic Stochastic Model Using Second-Order Monte Carlo Simulation

**FIGURE 7:**
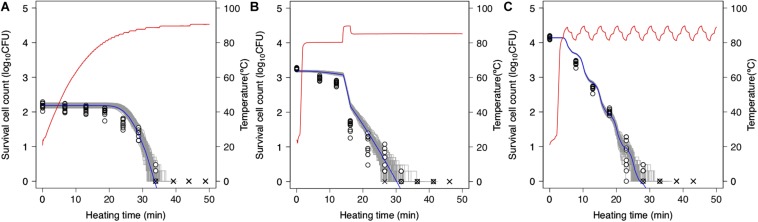
Predictions of second-order Monte Carlo simulation (gray) and dynamic kinetic model (blue) and changes in 10 replicates of observed survival *Bacillus simplex* spore counts (points) under each temperature profile (red curve) of slow come-up heating in pH 6.5 **(A)**, bumpy heating in pH 6.3 **(B)**, and waving in pH 6.4 **(C)**. ×represents no colonies were detected.

Simulations based on 1,000 repetitions of the secondary models derived from the non-parametric bootstrap methods were used to model the variation in bacterial reduction behavior, including the variability and uncertainty in the fitting model estimation (Figure 7). In the figure, the temperature profile and dynamical kinetic model predictions based on prior research (Peleg and Penchina, 2000; Campanella and Peleg, 2001) are shown as red and blue lines, respectively, while the gray lines indicate the results of 100 runs of simulated spore counts under non-isothermal inactivation. It is seen that the reduction rate follows the heating temperature, increasing and decreasing as the latter increases and decreases, respectively. Moreover, the predicted survival cell counts can describe the tendency of the observed bacterial behavior (0.12 < RMSE < 0.55). However, there were differences between observed and estimated values in some conditions. As described below, the cause of these discrepancies would be considered to be another factor that cannot be expressed in the 2DMC model. At small bacteria counts, there is a large variation in inactivation times until an arbitrary bacterial count (Figure 7). The variability in the time needed to reduce the surviving cell count to a specific level is increased in the range below 2 log_10_ CFU. The kinetic prediction passed through the center of the estimated range of survival spore count derived from the stochastic model, it is suggested that kinetic models tend to describe the average behavior of bacterial heterogeneity. The points in the figure indicate the observed surviving spore counts and are used to validate the 2DMC simulation model. Although the observed values in the region below 2 log_10_ CFU are within the 2DMC-estimated range, those above 2 log_10_ CFU are outside of the estimated range.

The 2DMC developed in this study gives the distribution derived from the bootstrap and reflects the stochastic process describing bacterial reduction. In this manner, it describes both the uncertainty in observed value and the variability in bacterial lethality. Although most previous stochastic models describing bacterial behavior were based on only variability (Corradini et al., 2010; Aspridou and Koutsoumanis, 2015; Abe et al., 2019; Koyama et al., 2019b) or uncertainty (Schaffner, 1994), some researchers have advocated using models that integrate variability and uncertainty estimation (Cassin et al., 1998; Nauta, 2000; Pouillot and Delignette-Muller, 2010; Abe et al., 2019). In this study, the variations of parameters were described with bootstrap while the randomness of dead cell counts were described with MCMC. The variations of parameters can describe the fitting uncertainty and the heterogeneity in individual cell thermotolerance; the randomness of dead cell counts can describe the true randomness in behaviors of bacteria population with exactly the same properties.

### Evaluation of the Validity of the Second-Order Monte Carlo Simulation

**FIGURE 8:**
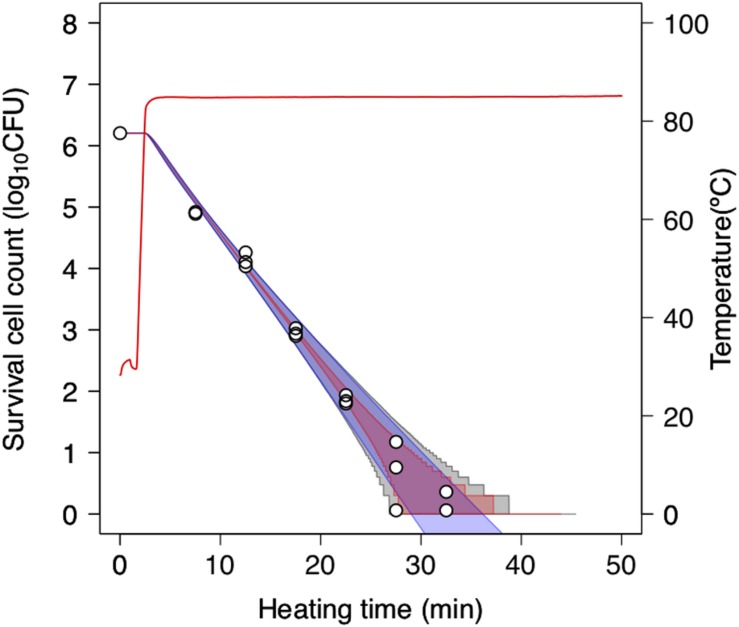
Comparison of 99% confidence intervals derived from only-bootstrap model (blue region), only-MCMC model (red region), and 2DMC models (gray region) during heating at 85°C in pH-adjusted TSB at pH 5.4. The data points indicate two or three replicates of observed values. The only-bootstrap model is derived from the dynamic kinetic modeling of 1,000 secondary-model sets using the bootstrap method, while the only-MCMC model is derived from a dynamic stochastic secondary model constructed without application of the bootstrap model.

To further assess the 2DMC approach, we compared it to “only-bootstrap” and “only-MCMC” models, i.e., to a dynamic kinetic model derived from 1,000 secondary model sets using the bootstrap method and a dynamic stochastic model derived from a secondary model without the application of bootstrapping, respectively. Figure 7 shows the 99% confidence intervals for the time required to obtain a specific decrease in the number of surviving cells under a thermal condition (pH 5.4, heating temperature 85°C) derived from the only-bootstrap (blue region), only-MCMC (red region), and 2DMC models (gray region). We compared the three models in terms of their confidence intervals. At surviving cell counts larger than 2 log_10_ CFU, the confidence intervals of the 2DMC model matched those of the only-bootstrap model, whereas those of the only-MCMC model were much smaller. At surviving cell counts of less than 2 log_10_ CFU, the confidence intervals of the 2DMC were larger than those of the other models. Furthermore, the confidence intervals of the 2DMC model corresponded to the variations in the observed values (Figure 8). These results further validate the use of a 2DMC model that describes both uncertainty and variability and suggest that a stochastic model combining multiple Monte Carlo simulations, such as the one used in this study, can accurately estimate cell reduction behavior that includes both variability and uncertainty.

### Potential of Adaptation for Other Bacterial Behavior Description

The 2DMC simulation model proposed in the present study (Figure 7) is based on the previously developed Weibullian dynamic models. One of the fundamental formulations used to develop the 2DMC, Eq. 6, is based on the previous kinetic Weibullian dynamic studies (Campanella and Peleg, 2001; Peleg and Normand, 2004; Corradini and Peleg, 2009). The applicability of the Weibullian dynamic kinetic model to other types of bacteria has been demonstrated in the previous studies, with the results of these studies confirming our description of bacterial reduction behavior under non-isothermal heating by an adaptation of conventional kinetic models. In addition to the conventional Weibullian dynamic models mentioned above, the proposed model enables to assess the variation in bacterial count during an actual thermal process. This suggests the possibility of developing a 2DMC model based on a database such as ComBase (Baranyi and Tamplin, 2004) for not only average behaviors but also variabilities and uncertainties of vegetative cells’ behaviors as well as spores, although the results would need to be validated. Generalizing the proposed model, the model can also be used to calculate the bacterial or sporular death behaviors including variation in real-time under existing or planned industrial thermal processes.

There is a possibility of application of the Markov property for other dynamic model than thermal inactivation. The proposed 2DMC model performs MC simulation by applying the Markov property, i.e. by using only the current state to predict the next state, with any other information about the past states disregarded (Durrett, 2011). This simulation approach, referred to as MCMC, is generally used for the simulation of stochastic processes with probability densities known up to a constant of proportionality (Geyer, 1992). In this paper, the Markov property is introduced in Eq. 10. Unlike the conventional stochastic Weibullian dynamic model (Corradini et al., 2010), which does not consider the influence of the randomness of instantaneous survival ratio, the proposed stochastic dynamic model takes both *S*_*(t)*_ and the instantaneous count of surviving bacteria *N*_*(t)*_ into account. From Eq. 5, as adapted from previous work (Mattick et al., 2001; Peleg et al., 2005), the randomness of the instantaneous survival ratio depends on the instantaneous values of *S*_*(t)*_. Considering the dependency to instantaneous *S*_*(t)*_, it is important to take an idea of the Markov property into account for not only in Weibull models but in any type of reduction model. In addition to reduction behavior, formulations based on the Markov property such as Eq. 10 can describe growth behavior because growth behaviors depend on the instantaneous bacterial counts. Thus, by applying MCMC it is possible to create multiple stochastic prediction models even under dynamic environments.

### Limitations of the Developed Modeling Procedure

The proposed dynamic model, however, might have some limitations in its ability to predict variations in the reduction behavior of arbitrary bacterial types. The proposed model ignores several factors, including the interactive influence between bacteria and thermal unevenness. As cell (and in this particular case spore) interactions are not taken into account in the developed modeling approach, it is possible that the proposed model cannot be used to predict bacterial population dynamics involving such interactions. As Eq. 10 describes change over an infinitesimal interval under any type of bacterial reduction model, it can be applied via Eq. 10 to express average bacterial behavior through a kinetic model. In this study, very small (100 μL) bacterial suspensions were tested to avoid uneven temperature conditions across the samples. Under conditions of actual thermal inactivation in food or beverages, however, there will be significant temperature unevenness as a result of heating, which should therefore be taken into account through the use of a temperature-predictive physical model established via the modeling of heat transfer with a fine mesh. By doing so, the problem of kinetic modeling of the interactive dependence among bacteria can be solved.

Another critical limitation with the proposed model is that it cannot completely express uncertainty and variability. The proposed 2DMC method attempts to use the bootstrap method to express uncertainty in the Weibull model parameter estimations, although it does not account for uncertainty related to experimental procedures (e.g., plate counting), model selection and other sources. The same applies for variability sources not explicitly taken into account in the present modeling approach such as intra-species (i.e., strain-to-strain) variability, other than true randomness and thermotolerance variability within a bacterial strain. The differences seen in Figure 7 between the estimated and observed surviving cell counts in the range above 2 log_10_ CFU demonstrate its failure to obtain accurate parameter values. This discrepancy arises from various factors in the creation of the primary and secondary models, and the development of a more robust dynamic model requires the selection of a more appropriate combination of these models. The proposed model is based on a conventional kinetic dynamic model, and the use of inappropriate secondary or primary models can therefore result in inappropriate dynamic predictions. In particular, the bootstrap process through which the model estimates the parameter distribution involves the selection of a most suitable value for each iteration via the least square method. However, the standard error of fitting using the least squares method was not considered in this study. In future applications of the proposed method, this could be addressed by resampling the prediction parameters based on a normal distribution with a standard error equivalent to that of the fitting to widen the parameter distribution and obtain a more accurate parameter value. In addition, Bayesian analysis might be useful in describing the uncertainties in the primary parameters. Given that the observed values used for validation will themselves have inherent and unavoidable uncertainties, full validation of the stochastic process model will require a method that can completely account for uncertainty in observed values.

## Conclusion and Future Perspectives

If the problems described above can be overcome, it would be possible to model the heterogeneity of bacterial behavior throughout the entire manufacturing process. A simulation model such as the one described in this study can be used to obtain the survival probability of bacteria populations and a distribution of surviving cell counts (Abe et al., 2019), which would aid in the risk assessment of food processing. Real food manufacturing processes inevitably have many variabilities and uncertainties. Describing all the variabilities and uncertainties using 2DMC or even 3DMC or higher-order simulations to obtain better-detailed assessments of heterogeneities in bacterial behavior will enhance the bacterial food safety in the manufacturing processes. Such models would also make it possible to determine the inactivation conditions of safety-guaranteed foods while better maintaining original food quality.

The results of this study demonstrate the potential of 2DMC simulation to describe bacterial inactivation behavior during non-isothermal inactivation with variability in individual cell heterogeneity and parameter uncertainty both taken into account. Comparison of the results of the 2DMC approach with models looking only at uncertainty or variability further revealed the importance of using a combined model. The 2DMC simulation model developed in this study should be useful in quantitative microbial risk assessment, aiding in the design of risk-based thermal processes for the efficient inactivation of bacterial spores, and ensuring food safety and quality with minimal negative impact on the sensorial attributes of foods.

## Data Availability Statement

All datasets generated for this study are included in the article/supplementary material.

## Author Contributions

HA, KK, and SK designed the study and wrote the manuscript. HA, KT, and SD performed the study and analyzed the data.

## Conflict of Interest

The authors declare that the research was conducted in the absence of any commercial or financial relationships that could be construed as a potential conflict of interest.

## Supplementary Material

Data Sheet S1 Dataset and the R script for the calculation.

## Abbreviations

2DMC: Second-order Monte Carlo
CFU, colony forming unit MC: Monte Carlo
MCMC: Markov chain Monte Carlo
PCR: polymerase chain reaction
QMRA: Quantitative microbial risk assessment
root mean square error: RMSE
RTE: ready-to-eat
TSB: tryptic soy broth.
